# The SGC beyond structural genomics: redefining the role of 3D structures by coupling genomic stratification with fragment-based discovery

**DOI:** 10.1042/EBC20170051

**Published:** 2017-11-08

**Authors:** Anthony R. Bradley, Aude Echalier, Michael Fairhead, Claire Strain-Damerell, Paul Brennan, Alex N. Bullock, Nicola A. Burgess-Brown, Elisabeth P. Carpenter, Opher Gileadi, Brian D. Marsden, Wen Hwa Lee, Wyatt Yue, Chas Bountra, Frank von Delft

**Affiliations:** 1 Structural Genomics Consortium (SGC), Nuffield Department of Medicine, University of Oxford, Roosevelt Drive, Oxford OX3 7DQ, U.K.; 2 Department of Chemistry, Chemistry Research Laboratory, 12 Mansfield Road, Oxford OX1 3TA, U.K.; 3 Diamond Light Source Ltd., Harwell Science and Innovation Campus, Didcot OX11 0QX, U.K.; 4 Department of Molecular and Cell Biology, Henry Wellcome Building, Lancaster Road, Leicester LE1 7RH, U.K.; 5 Target Discovery Institute (TDI), Nuffield Department of Medicine, University of Oxford, Oxford OX3 7FZ, U.K.; 6 Kennedy Institute of Rheumatology, Nuffield Department of Orthopaedics, Rheumatology and Musculoskeletal Sciences, University of Oxford, Roosevelt Drive, Oxford OX3 7FY, U.K.; 7 Department of Biochemistry, University of Johannesburg, Auckland Park 2006, South Africa

**Keywords:** FBDD, Fragment screening, Genetic hits, SBDD, SGC, Structural Genomics

## Abstract

The ongoing explosion in genomics data has long since outpaced the capacity of conventional biochemical methodology to verify the large number of hypotheses that emerge from the analysis of such data. In contrast, it is still a gold-standard for early phenotypic validation towards small-molecule drug discovery to use probe molecules (or tool compounds), notwithstanding the difficulty and cost of generating them. Rational structure-based approaches to ligand discovery have long promised the efficiencies needed to close this divergence; in practice, however, this promise remains largely unfulfilled, for a host of well-rehearsed reasons and despite the huge technical advances spearheaded by the structural genomics initiatives of the noughties. Therefore the current, fourth funding phase of the Structural Genomics Consortium (SGC), building on its extensive experience in structural biology of novel targets and design of protein inhibitors, seeks to redefine what it means to do structural biology for drug discovery. We developed the concept of a Target Enabling Package (TEP) that provides, through reagents, assays and data, the missing link between genetic disease linkage and the development of usefully potent compounds. There are multiple prongs to the ambition: rigorously assessing targets’ genetic disease linkages through crowdsourcing to a network of collaborating experts; establishing a systematic approach to generate the protocols and data that comprise each target’s TEP; developing new, X-ray-based fragment technologies for generating high quality chemical matter quickly and cheaply; and exploiting a stringently open access model to build multidisciplinary partnerships throughout academia and industry. By learning how to scale these approaches, the SGC aims to make structures finally serve genomics, as originally intended, and demonstrate how 3D structures systematically allow new modes of druggability to be discovered for whole classes of targets.

## Introduction

One of the main challenges in the biomedical sector, whether industrial or academic, is how to obtain strong validation (or devalidation) of disease linkage for potential targets of interest. Large genomic studies [1,2] are rapidly yielding long lists of such targets, yet translating this information into testable hypotheses in disease-relevant models remains a generally intractable challenge. Nevertheless, these targets rightly continue to be seen as pointing the way to where to seek drugs for diseases of unmet need.

The route from genetic hit to small molecule treatment is long, and traverses research communities with divergent cultures and very different languages. Thus biologists, to validate hits, will quickly turn to phenotypic approaches such as RNAi, siRNA, mouse knockouts, ChIP-seq and latterly of course CRISPR. Such data are certainly crucial for validating a target, yet they also come with many caveats intrinsic to the techniques, and more importantly do little on their own to illuminate the basis for the disease linkage. In particular, developing small molecule inhibitors from phenotypic activity alone, while certainly possible and frequently advocated, does present significant hurdles. Most dauntingly, identifying starting compounds usually requires high-throughput screening (HTS) with very large compound libraries. This not only involves very significant infrastructure and costs, but also more seriously, carries a high risk of false positives and thus demands extensive and cautious follow-up effort to confirm and then pursue hits – especially if the target or mechanism of action is not known, which is usually the case and challenging to elucidate. Furthermore, compounds need highly specific compound properties, for example cell permeability, greatly limiting the scope of available reagents.

The more reductionist approach is to isolate the gene product (or a fragment) recombinantly, and use this to discover biochemical activity and develop compounds that bind and potently inhibit activity (‘chemical matter’). Such compounds, even when a long way from clinically relevant, are powerful tools for probing the physiological role of the target gene *in vivo*, provided they are sufficiently potent, specific and cell penetrant, hence their designation as ‘*tool compounds’* or ‘*chemical probes*’ [3]. The power of the concept has long been embraced in industrial drug discovery, and over the last decade has also driven considerable academic activity [4], not least on the part of the SGC [5].

The challenges around isolating the protein and developing meaningful biochemical assays are considerable; at the same time, they are the bread-and-butter of conventional structural biology, which has witnessed tremendous improvements in the last two decades, including extensive technical contributions arising from the various structural genomics initiatives.

More relevant here is the problem of developing chemical matter. Drug-like chemical space, defined as the set of all molecules with oral drug-like properties (size, hydrogen bonding, lipophilicity), is estimated to comprise 10^23^–10^60^ molecules, much larger than all the known organic molecules (∼10^8^) [6]. Therefore, it is assumed that for the druggable genome, there must exist at least one small molecule ligand that potently binds each of the estimated 4500 potential targets [7]; however, searching such a large space by even simplistic and thus inaccurate computational algorithms is inconceivable.

Instead, a multidisciplinary approach is required, whereby initial hit compounds are identified followed by iterations of ligand design, synthesis and testing for potency, during which medicinal chemists seek to improve potency by modifying the previously best compounds. Hit compound searching can be done via a variety of biochemical, biophysical and computational methods [8,9]; however, *a priori* knowledge of the target family’s ligand preferences greatly improves this approach and has hugely accelerated the discovery of new ligands for well-studied target classes such as kinases [10] and bromodomains [11]. Similarly, knowledge of the target protein’s 3D structure greatly speeds up both hit searching and evolution, known as structure-based lead design (SBLD) – or, more loosely, structure-based drug design (SBDD).

The approach is particularly challenging for the singleton targets and novel target classes unearthed by the flood of data from genomics, proteomics, metabolomics and other omics approaches, since there are no prior target/ligand 3D structures and pharmacology data to consult. Here, unbiased approaches are required for finding initial hit compounds, and HTS of large (0.5–1 million) compound libraries against *in vitro* assays have been the default technique, despite the following drawbacks. For a start, the need for an assay requires functional knowledge of the target, which may be unknown. Moreover, apart from the cost of infrastructure, the compounds libraries are invariably biased towards historic drug discovery efforts, or else to combinatorial chemistry that favours robust and short synthetic routes. Even the largest libraries of sufficiently complex compounds (17–34 heavy atoms) cover only a vanishingly small percentage of chemical space (compare the 34.5 million compound Enamine realDB (REAL DataBase – Enamine, http://www.enamine.net/index.php?option=com_content&task=view&id=8) library of synthetically tractable compounds to the 166 billion enumerated compounds in GDB-17 [12]). Finally, the nature of the biological assays used makes it very difficult to identify ligands with novel (non-competitive) modes of action.

By contrast, fragment-based lead discovery (FBLD) has established itself as a major method for finding hits for novel targets: fragments are defined as very small molecules (6–18 heavy atoms), and thus even a small library (e.g. the 300 compound library by Zenobia (Zenobia Fragments, https://www.zenobiafragments.com/products)) provides far better coverage of chemical space and gives a much higher hit rate than HTS [13]. Highly sensitive biophysical techniques are required to identify the weak fragment binding events; most commonly, solution-based techniques (e.g. NMR, SPR) are used as primary screen. The most challenging aspect of FBLD is that significant expertise and resource are needed to turn a fragment hit into a potent and selective molecule. On the other hand, a major strength is that because molecules are evolved from the start, very specific modes of action can be targeted. Finally, for novel targets, establishing their unknown biochemical function is greatly aided by the availability of a potently binding molecule.

## The problem

Given the large resource of interesting untapped genomic data on the one hand, as well as the existence of well-established approaches to obtaining chemical matter, it is worth asking why we are not seeing a flood of compounds identified and progressed against such novel targets. Several broad trends seem relevant.

The industrial community, while it has the experience to do the lengthy and intellectually challenging work of target validation, is increasingly looking to the academic and biotech sectors to derisk targets. In contrast, the academic community is generally either inexperienced, underfunded or inefficient because the piecemeal nature of its funding makes it tremendously difficult to assemble the requisite skillset and critical mass; while those labs to which this does not apply, such as various larger size academic drug discovery units, tend to apply the same rigorous selection criteria to their targets as industry. Finally, the biotech sector does move very fast indeed, but the nature of its business model means that its discoveries do not lend themselves to crowd-sourcing, but are also subject to investment priorities that are decoupled from the needs of a broader scientific problem.

## Target Enabling Packages (TEPs)

The considerations above convinced the SGC that a strategic effort was needed to develop systematic approaches to bridging the gap between genomic disease linkage and small molecule discovery, and thereby help to understand disease biology. Funded by a Wellcome Trust strategic award, this SGC programme aims to develop reagents, protocols and data, collectively called a Target Enabling Package (TEP) (SGC | Target Enabling Packages (TEPs), http://www.thesgc.org/tep), which will accelerate the assessment of genetic hits as potential drug targets. In its current incarnation, a TEP minimally comprises:

- A protocol to express and purify the protein (gene product)
- A robust activity assay
- A crystal structure and robust crystallization protocol
- Convincing chemical matter, i.e. either potent or else observed bound to a crystal structure, showing clear elaboration possibilities

Focusing on genetic hits from metabolic- and inflammatory diseases, neuro-psychiatry and cancer, the current TEP initiative seeks to engage with the global community to generate high quality reagents around these gene products. By bringing together experts in diverse fields (biology, diseases, structural biology, medicinal chemistry), it aims to stimulate the global community to invest in novel potential targets identified from genetic hits, deorphanizing their biological function and thereby validating or devalidating their tractability for small-molecule targeting. Beyond the role of the TEP initiative in translational activities, it is anticipated that the TEPs will have a major contribution in deciphering new biology and disease-associated pathways.

Central to the initiative is that it is embedded in a community of interested scientific networks. Targets are actively recruited from and nominated by disease experts and geneticists, and are prioritized in discussion fora, the four disease-area Target Prioritization Networks. During development of individual TEPs at the SGC, nominators are consulted as collaborators, to ensure relevance of the outputs, and to help deorphanize the targets. Finally, a TEP Evaluation Group (TEPEG) annually considers and approves or rejects proposed TEPs, judging how useful they could be for actual drug discovery programmes and/or for the disease biology community. Crucially, after approval the work is freely released to the community, to ensure maximum take-up.

The selection of targets is inevitably a major focus of activity, and the discussions are deliberately extensive. Nevertheless, we are seeking to converge on selection criteria that can be disseminated in time, as part of the overall TEP framework. Currently they include: the robustness of the available disease association; the significance of the disease phenotype; the biological plausibility; the need for biochemical and structural reagents; the feasibility of producing high quality work with our methodologies; and the prospects for exploiting TEPs. The latter includes druggability, though that is now a looser constraint, since it is in fact better probed through unbiased XChem fragment screening and follow-up chemistry (below).

The project is both ambitious, seeking to generate over 24 TEPs, yet also a pilot, considering how many strong genetics hits are actually emerging with disease linkage. Its desired impacts include: to generate starting point for projects in the pharmaceutical industry and in academic settings; to catalyse novel translational research and drug discovery; to participate in functional deorphanization and disease mechanisms; and to provide starting points for probe development. Annual symposia aim to nurture the exchange of knowledge and reagents between the diverse fields of experts.

## Technologies

**Figure 1:**
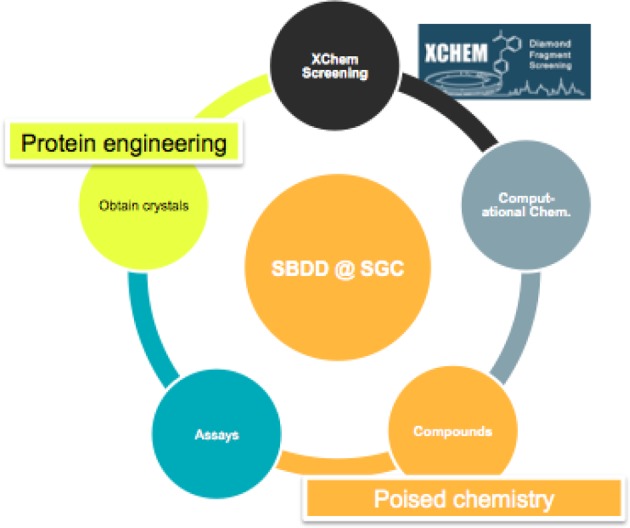
Technologies which required development to ensure delivery of the promised TEPs

Delivering the chemical matter for TEPs required for the development of new technologies in three areas, illustrated in Figure 1: a new version of fragment screening by X-ray crystallography (XChem), with an order-of-magnitude improvement in throughput; protein engineering to ensure reliable crystal growth for the thousands of crystals needed for X-ray screening; and finally, novel techniques for rapidly synthesizing follow-up compounds.

### Protein engineering

Although it is the most commonly used technique for determining a protein’s structure, X-ray crystallography is entirely reliant on the production of suitable protein crystals. Finding these crystallization conditions is challenging anyway, but ensuring they are reproducible is invariably more problematic, as crystallographers tasked with supporting ligand discovery know very well. The XChem experiment requires thousands of crystals per target; and furthermore, availability of alternative crystal forms for the same protein are often essential, since crystal packing can occlude the desired binding site.

The most important determinant of robustness of the crystal system is the exact sequence of the expressed protein, and therefore we developed an experimental protocol that enables taking dozens of variants from expression through purification and concentration all the way to crystallization, within a week and minimal effort. This PREPX protocol (manuscript in preparation) has proved a workhorse technology, as it allows the variants to be evaluated by the metric that matters, namely their ability to crystallize. To date, it has yielded crystals suitable for fragment screening for 7 of the 10 of targets it was used for. Thus, not only can the boundaries of the gene constructs be efficiently explored, but also the various kinds of surface mutations to improve the likelihood of crystal packing. In conjunction, what proved crucial was developing informatics tools for helping to design the variations to be introduced.

The approach requires good expression and solubility, which are historically seen as proxies for tractability for crystallization, but are not in fact the same thing. Solubility and expression are properly explored by protocols previously reported, such as parallel 1 ml of test expression [14] or various fusion constructs, especially MBP [15]. Usually, expression can be optimized for one variant and then applied to all others. The protocol is optimized for *Escherichia coli* expression, where 24–48 constructs can be handled in a single round; work on a protocol for insect cell expression is in progress, but it already supports 6–12 constructs simultaneously. The general principles of streamlining parallel protocols are certainly applicable to other expression systems too, but invariably the details matter, and these will be addressed as driven by the priority of TEP targets refractory to expression in our current systems. A particular bias that PREPX mitigates is for protein chemists to pick their construct of choice too early, based on test expression results. We now routinely test all available constructs for crystallization, prompted by a series of dramatic early success stories.

### XChem: fragment screening by crystallography

Due to its sensitivity and direct information on protein–ligand interactions, X-ray crystallography is an invaluable and widely used method in SBLD and FBLD; indeed, X-ray crystallography was one of the first methods to be used, and is the most directly informative [16]. Nevertheless, it has not been widely used for primary screening, since the experimental overheads have historically been too high. Outside of specialized groups, the method is considered low-throughput, suited to tens of experiments (single-compound soaks) at a time.

The SGC partnered with Diamond Light Source synchrotron helping it to establish the pioneering XChem facility at beamline I04-1, where truly high-throughput crystallographic fragment screening is now available.   A highly streamlined workflow allows up to 1000 compounds to be screened individually against a single target in less than a week (including 36-h unattended beam time).  The workflow covers soaking, harvesting, automatic data collection and data analysis; fragment libraries are available.

A number of significant developments underpin the XChem facility. (i) Acoustic liquid handling delivers compounds to crystals with high speed and accuracy (900 compounds within 30 min [17]). (ii) Semi-automated crystal harvesting with the newly developed Shifter robot enables even novice users to process ∼130 crystals per hour. (iii) A highly automated and stable beamline (I04-1) can now collect over 700 X-ray diffraction datasets in 24 h. (iv) Advanced high performance computing infrastructure for image analysis can process the millions of diffraction images fully automatically. (v) We developed tools that greatly simplify interpretation of weak density (PanDDA [18]), and streamline the refinement of hundreds of crystal structures (XChemExplorer [19]). Furthermore we have been working with the wwPDB to deliver streamlined deposition of structures: most recently we deposited >800 datasets to the Protein Data Bank (PDB) in a single batch, and in future, there will be support for analysis and presentation of hit results, and deposition of hit structures for all XChem users.

The XChem experimental design aims to maximize the hit-rate and thus the information gained from screening a target biomolecule. Thus, singleton soaking is the default, to maximize the effective concentration of each compound and simplify analysis of density. This is in contrast with the traditional approach of using cocktails of 4–10 compounds, a historic necessity of low throughput. In addition, through careful calibration, compound concentrations are maximized so that DMSO concentrations of 50% can be achieved, using 500 mM compound concentrations. A mark of the effectiveness of the technique is that a 2016 audit revealed that of 20 targets screened, all had yielded hits, with almost 800 hits in total. The facility is open to users from around the world and has also been instrumental in the TEPs delivered to date.

The nature of the experiment places fairly stringent demands on the crystal system. Firstly, it is essential to have a crystallization protocol that allows crystals to be readily reproduced on demand, not only at home but also at Diamond. The most reliable yet underused technique is seeding with seed stock solutions, and the perfect protocol entails sending three vials of frozen protein, seed stock and mother liquor, along with a detailed protocol already tested at home to emulate transit. Currently, an additional constraint is ability to grow in sub-microlitre sitting drops in microtitre plates, although other formats will be supported in future, in particular LCP. What is not important is for crystals to grow in *every* drop, thanks to the random access capabilities of the robots.

The final crucial requirement is consistency of diffraction: since duplicates are too expensive in beamtime, cryoprotection and harvesting protocols are needed under which every crystal diffracts to a roughly similar resolution. Where this is not achievable, different crystal forms need to be sought, or the protein reengineered. It is less crucial to have very high resolutions: thanks to the PanDDA [18] algorithm, systems as poor as 2.5 Å have yielded meaningful hits to date.

The PanDDA [18] algorithm has had two other, related consequences. Firstly, because it is able to extract clear density even for low occupancy binders, the upstream soaking and diffraction experiment does not apparently require duplicates, as was historically common for crystallographic fragment screening. Secondly, it enables binding events to be identified with high confidence, eliminating the need for either lengthy density interpretation or even repeating the experiment; indeed, false positives appear to be largely absent, in contrast with the case in conventional electron density. Hit rates (total number of events) lie typically in the 1–10% range.

### Rapid follow-up chemistry

What the PanDDA algorithm cannot do, is help to decide which hits are chemically and biologically interesting. For instance, part of its power lies in identifying binding events across the protein in a site agnostic manner; clusters of hits might indicate allosteric opportunities, but proving this would require progressing some hits to true potency followed by thorough biochemical analysis, an unfundable proposition. Analysing the wealth of information is such a challenge that we have adopted the aphorism that a hit is anything a chemist is prepared to work on; and early on it was clear that more scalable approaches would be required.

The major challenge in all fragment screening is that the ligand hits, due to their small size, have fewer molecular interactions with their target and consequently much weaker binding than HTS hits (*K*_D_ 10–1000 μM vs 0.1–10 μM), and thus require more optimization to get to useful potency.  At the same time, this requirement also gives the medicinal chemist the opportunity to optimize for other oral drug attributes (polarity, metabolic stability, selectivity) concurrent to potency improvement, in contrast with HTS hits which often come with imperfections inherent in their structure which can be difficult to design out of larger molecules.

Because fragment libraries need only be small (10^3^–10^4^ compounds), they can be assembled to exacting standards. Traditionally, fragments have been selected for library inclusion based on physicochemical properties and diversity, but recent years have seen efforts to create libraries with unique structures such as 3D-character [20] and synthetic enablement. Murray and Rees highlighted the importance of fragments which can be expanded synthetically at every vector position of a fragment hit [21].

**Figure 2:**
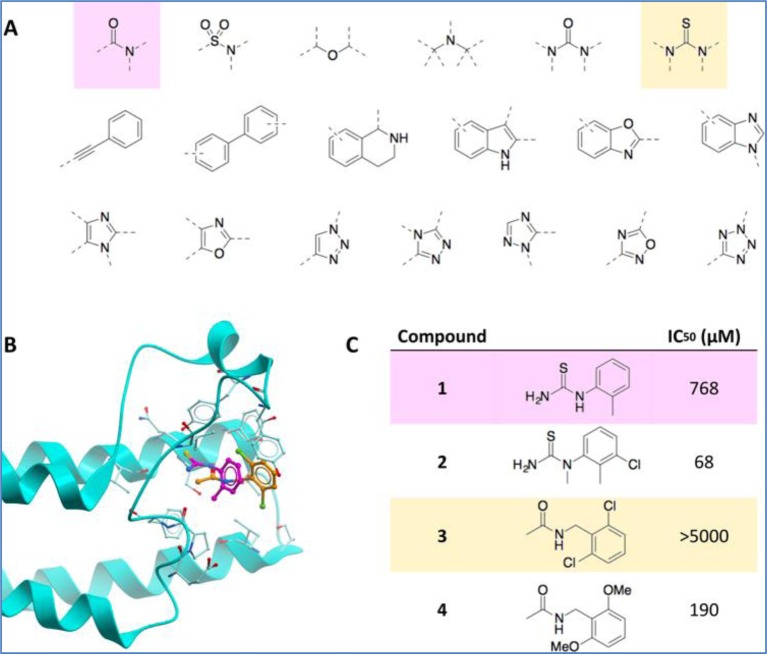
Design and screening of a synthetically enable, ‘poised’ fragment library (DSPL) identified two hits for the atypical, second bromodomain of PHIP (**A**) All fragments in DSPL contain drug-like cores that can rapidly be constructed from robust reactions with populous building blocks. PHIP hits substructures highlighted. (**B**) Crystal structures of hits 1 (lavender) and 2 (peach) in the second bromodomain of PHIP. (**C**) Synthesis of analogues of hits 1 and 2 gave compounds 3 and 4 with measurable IC50’s.

Orthogonally, we designed a ‘poised’ fragment library [22], comprising compounds selected specifically to allow (i.e. be ‘poised’ for) rapid, cheap but flexible follow-up chemistry. All members of the library, if discovered as a hit, can be rapidly expanded via robust parallel chemistry to give analogues for evaluation.  The content of the library is openly available under the name Diamond SGC Poised Library (DSPL). Its first proof of principle was showing how crystallographic fragment hits could be rapidly optimized for the second and atypical bromodomain of PHIP, a cancer target (Figure 2). We are now seeking to apply this approach to all targets, to the extent that there is funding available: our experiences on a few recent targets indicate that hits can be progressed from unmeasurable to low micromolar potency, and thus somewhat validated, at a reagent cost of £5–10k.

### Partnerships

As Figure 1 illustrates, there remains much room for further technological step changes. Here too the SGC is relying on partnerships across academia and industry, two of which deserve mention.

In the area of computational chemistry, we have convened a nascent Collaborative Computational Project, named CCP-CompMedChem (CCP-CMC) (http://www.ccp-cmc.org/). CCP-CMC is modelled on the long-lived and highly successful CCP4 [23] (crystallography), and is a collective of ∼50 academic and industrial scientists involved in developing and using computational methods in drug discovery. The long-term aims are to give naïve users access to best practices and easy-to-use tools for computational chemistry, and provide a route-to-users for academic tools and technologies. Through the open-sourced SQUONK platform (https://squonk.it/) and containerization technologies (Docker; https://www.docker.com/), the tools can be made freely available to the community. The various TEP projects will provide an exceptionally useful set of data for bringing such tools to maturity.

In the area of synthetic and medicinal chemistry, we are seeking to engage the synthetic chemistry community to expand the repertoire of reactions captured in the poised library. Through Ox XChem, a pilot project (https://xchem.github.io/oxxchem/), we are establishing a screening library of fragments derived from UK academic groups which are available for screening on the XChem platform (and published openly). In future, such a library would credibly speak to the Astex challenge [21], once again enabled through demonstration on the openly available results from TEP targets.

## Outlook

While the TEP project has specific goals around particular disease areas, the bigger ambition is to explore in greater depth what it entails to do structural biology that is aimed at drug discovery, and ensure the necessary techniques are widely available. A 10-year vision is that no crystal structure is complete without a careful analysis of the target’s disease linkage, a fully analysed fragment screen, and a series of follow-up compounds with demonstrated potency and rationalized SAR. Naturally costs will need to drop precipitously for this to be possible at the scale of output of even current structural biology; yet this is not inconceivable, considering how rapidly crystallography itself has changed in last 10 years.

Variations of the TEP approach certainly exist worldwide; what we are trying to do is to bring this together into a coherent and normalized strategy and articulate best-practice. We also envisage an evolution in the TEP concept; thus for instance it is already clear that establishing a biophysical assay for assessing low potencies is vital. Equally, the inclusion of biological reagents (e.g. CRISPR or siNRA) specifically developed to probe a given linkage, would be a powerful enabler, especially if freely available.

Of course, TEPs on their own, even the advanced incarnations, will not be sufficient to unravel disease biology: use of even *bona fide* probe molecules present pitfalls that demand experience and caution [24], and having TEP chemical matter in the public domain will inevitably lead to inappropriate use and overinterpretation of data. Nevertheless, such risks are inherent in all science, so as long as suitably prominent health warnings are attached and education of the end user community continues apace, the benefits of accelerating probe development and making it more broadly feasible by generating more raw material and starting points, far outweigh the risks.

Finally, it is clear that public repositories will need to evolve quite dramatically to capture the ensemble of information that comprises a TEP. Repositories like the PDB would be an obvious home, but because they entail reagents as well as data, TEPs will require new linkages between public repositories and commercial vendors. It is strategically funded efforts like the one described here that seem most likely to drive and mediate such discussions.

## Summary

- It remains difficult to progress from genomic disease linkage data to small molecule discovery.
- The SGC’s TEP project seeks to bridge this strategic gap in the biomedical landscape by engaging with the relevant communities, generating reagents and data and placing them in the public domain.
- These Target Enabling Packages represent a new paradigm in structural biology aimed at drug discovery.

## Abbreviations

FBLD: fragment-based lead discovery
HTS: high-throughput screening
SBDD: structure-based drug design
SBLD: structure-based lead design
SGC: Structural Genomics Consortium
TEP: Target Enabling Package
